# Designing tensile ductility in metallic glasses

**DOI:** 10.1038/ncomms3158

**Published:** 2013-07-17

**Authors:** Baran Sarac, Jan Schroers

**Affiliations:** 1 Department of Mechanical Engineering and Materials Science, Yale University, 15 Prospect Street, BCT Room 217, New Haven, Connecticut 06511, USA

## Abstract

Effectiveness of a second phase in metallic glass heterostructures to improve mechanical properties varies widely. Unfortunately, methods to fabricate such heterostructures like foams and composites do not allow controlled variation of structural features. Here we report a novel strategy, which allows us to vary heterostructural features independently, thereby enabling a systematic and quantitative study. Our approach reveals the optimal microstructural architecture for metallic glass heterostructures to achieve tensile ductility. Critical design aspect is a soft second phase, which is most effective when spacing between the second phase assumes the critical crack length of the metallic glass. This spacing should coincide with the second phase’s size, and beyond, the specific second phase morphology of the heterostructure is crucial. These toughening strategies are only effective in samples that are large compared with the spacing of the second phase. The identified design aspects provide guidance in designing tensile ductility into metallic glasses.

## Abstract (web summary)

Metallic glasses are normally stronger than their crystalline counterparts, but not good at deforming under tensile stress. Sarac and Schroers test the ductility of metallic glasses in a precisely engineered microsystem and identify an ideal heterostructure with maximized strength and toughness.

## Graphical abstract

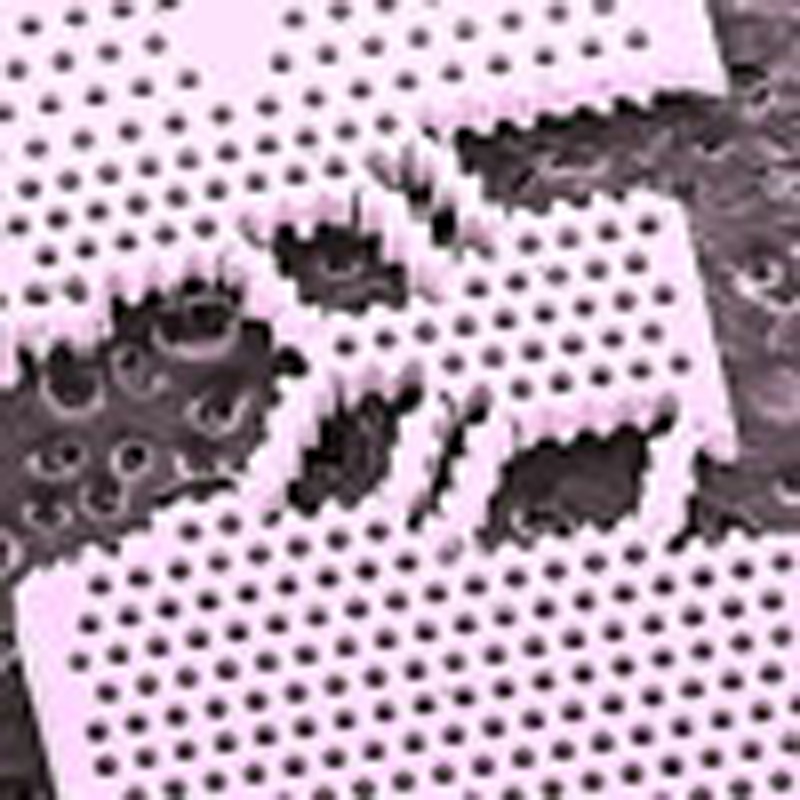

Despite high strength and elasticity combined with plastic-like processing ability, wide spread proliferation of metallic glasses (MGs) in structural applications has been stymied by their lack of tensile ductility^1^. Upon yielding, shear strain in MGs is highly localized into narrow, ~10-nm-wide bands. The development of such shear bands in MGs does not necessarily result in fracture. For example, when shear bands are spatially confined under certain loads and in small sizes, global plasticity enabled by the formation of a large number of shear bands has been observed^2^,^3^,^4^,^5^,^6^,^7^,^8^,^9^,^10^,^11^,^12^,^13^,^14^,^15^. In compression and bending, multiple shear band formation without crack formation has been reported^5^,^6^,^11^,^13^,^16^,^17^,^18^. In nano-sized samples, it has been argued through a Griffith-like criterion that the elastic energy release does no longer offset the energy barrier to form a shear band^10^. Hence, on such a small scale, a deformation mechanism not relying on shear bands can result in tensile ductility^8^,^10^,^12^,^19^.

One emerging strategy to couple the attractive properties of MGs with plasticity is to introduce a second phase, which reflects and/or absorbs shear bands^2^,^4^,^6^,^9^,^20^,^21^,^22^,^23^,^24^,^25^. It has been suggested that this strategy should be particularly successful when spacing of the second phase coincides with the critical crack length of the MG matrix phase^9^. However, verifying this hypothesis as the origin of the toughening mechanism has been challenging as fabrication methods of MG composites and foams are complex, and do not allow independent and systematic variation of microstructural features, such as phase spacing, size, shape and volume fraction.

Here, we report a novel approach that allows us to completely independently vary microstructural features with high precision, and determine their effect on mechanical properties. Using this systematic approach, we precisely determine that ductility increases with increasing the soft second phase spacing up to a spacing comparable to the critical crack length of the matrix phase. Furthermore, we emphasize the importance of morphology, and identify and quantify the ideal heterostructure, which maximizes strength and toughness. The fracture strength of the optimized MG heterostructure almost reaches to the fracture strength of the monolithic MG while fracture strain and overall toughness of the MG heterostructure become 3.75 and 5 times higher than that of the monolithic MG, respectively. Our approach reveals the microscopic mechanism that results in tensile ductility of MG heterostructures, and provides guidance to design tensile ductility into MGs.

## Results

### Fabrication of MG heterostructures

**Figure 1:**
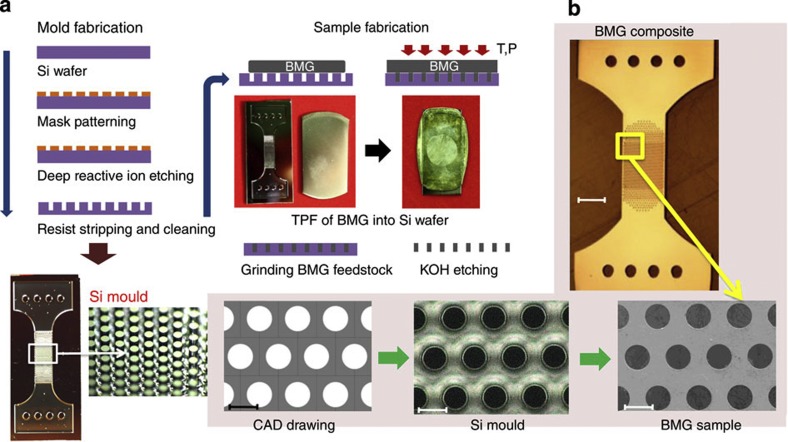
Fabrication steps of MG heterostructures. (**a**) MG (in other words, bulk metallic glasses -BMGs) heterostructures are synthesized through are synthesized through Si lithography to create negative moulds. Such moulds are subsequently replicated through thermoplastic forming (TPF)-based compression moulding. Excessive material is removed by sanding/polishing/thermoplastic hot scraping, and the sample is released from the mould by selective KOH etching. (**b**) MG heterostructure realized in a tensile test sample, and close-up image of its gauge section (scale bars 3 mm). Comparison of the Si mould with the original CAD drawing reveals the control and precision of our process (scale bars 100 μm).

Our method is composed of a two-step fabrication method, fabrication of a mould and replication of this mould with a MG (Fig. 1a). Silicon lithography is used to fabricate the moulds, and subsequent deep reactive ion etching allows to achieve deep cavities in the mould in excess of 400 μm for the required sample depth. Fabricated moulds comprise the (negative) heterostructure, which is embedded into the gauge section of the tensile test samples. The moulds are replicated with Zr_35_Ti_30_Cu_7.5_Be_27.5_ MG former through thermoplastic forming-based compression moulding^26^,^27^,^28^,^29^, and the fully amorphous state of these heterostructures is subsequently confirmed by differential scanning calorimetry (DSC) and X-ray diffraction (XRD). Noteworthy is the precision of this technique (Fig. 1b), where features of the CAD drawing are replicated into the MG heterostructure through the Si mould with a precision better than 1 μm.

### Mechanical characterization of MG heterostructures

**Figure 2:**
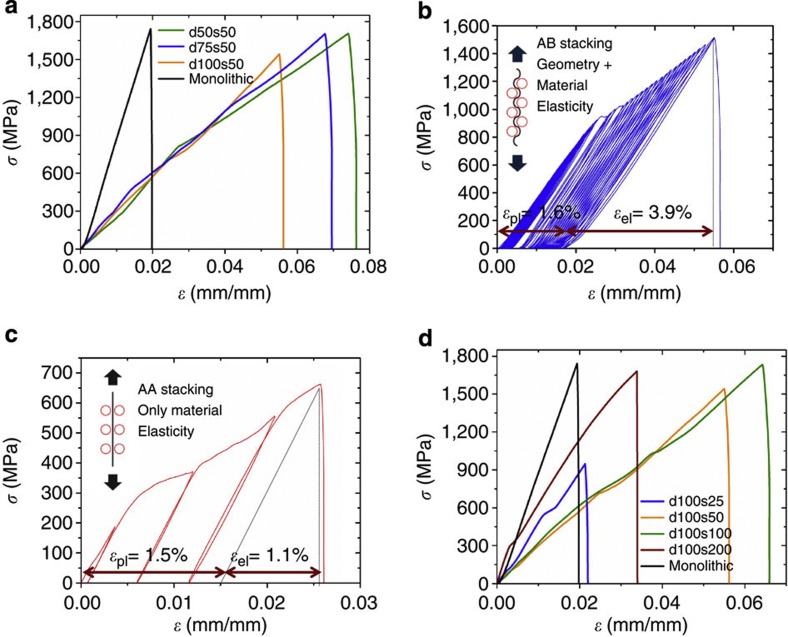
Quasi-static tensile characterization of MG heterostructures. Nominal fracture strength of the samples is calculated from the effective area. (**a**) Heterostructures with various pore sizes compared with monolithic MG. Increased cyclic loading of MG heterostructures with *d/s*=2. (**b**) AB stacking reveals 3.9% elastic deformation and 1.6% plastic deformation. (**c**) The amount of deformation in AA stacking is 1.1% elastic and 1.5% plastic. (**d**) Tensile ductility and strength increases with increasing pore spacing until this spacing exceeds the plastic zone size.

Tensile stress–strain curves for various MG heterostructures, and as a comparison, for a monolithic MG sample, are shown in Fig. 2a. The monolithic MG fabricated with our method exhibits an elastic strain limit of 2% and yield strength of 1,750 MPa, which is almost identical to the elastic strain limit of 2% and yield strength of 1,765 MPa, typically measured in the as-cast state of bulk monolithic rod-shaped samples in tension^30^. These almost identical values confirm that our fabrication method (Fig. 1) itself does not affect the mechanical properties. MG heterostructures with specific microstructural architecture exhibit a remarkably different mechanical response. For a microstructural architecture with pore diameter (*d*) and spacing (*s*) of 50 μm, the fracture strain increases dramatically to *ε*_f_≈7.5%. At the same time, Young’s Modulus of the heterostructure decreases, whereas the nominal fracture strength, *σ*_f_, remains relatively unaltered.

In order to deconvulate elastic and plastic deformation, we carried out cyclic loading with gradually increasing loads (Fig. 2b). This figure shows the contribution of elastic/plastic components of MG heterostructures until fracture under uniaxial tension, which changes with second phase morphology. A heterostructure with *d*=100 μm and *s*=50 μm was selected over *d*=*s*=50 μm heterostructure for sample preparation convenience. We found that of the 5.5% strain to failure, ~70% (3.9% overall) corresponds to elastic and ~30% (1.6% overall) corresponds to plastic deformation. The large elastic deformation originates from the AB pore-stacking order. According to cyclic results with different *d* and *s* (not shown here), the elastic to plastic deformation ratio remains relatively unchanged, which is estimated to show around 2.6% plastic and 4.8% elastic deformation for *d*=*s*=50 μm heterostructure. A heterostructure with the same pore diameter and spacing (*d*=100 μm, *s*=50 μm) but AA stacking exhibits a significantly lower strain to failure of 2.6%, where only ~40% (1.1% overall) deformation is elastic (Fig. 2c). Therefore, the geometrical effect caused by AB stacking increases the elasticity, and as a result, enhances the plastic deformation of the MG heterostructure.

When comparing MG heterostructures with identical pore size, but increasing pore spacing from 25–100 μm, both *σ*_f_ and *ε*_f_ increases. However, when the spacing exceeds 100 μm, both values drop dramatically (Fig. 2d). The highest performance in terms of *σ*_f_ and *ε*_f_ is present in heterostructures with a spacing between 100 μm and 200 μm. This spacing is close to the critical crack length, *a*_c_≈120–260 μm, of Zr_35_Ti_30_Cu_7.5_Be_27.5_, which is calculated according to a (1/π) (*K*_C_/*σ*)^2^ (dimensionless factor is taken as 1 for circular pores), with *σ*=1,750 MPa, and *K*_C_~35 MPa m^1/2^ (unpublished data)–50 MPa m^1/2^ (ref. 31). Precise data for *K*_C_ are rare for MGs, which is believed to originate from difficulties in sample preparation and a high notch radius sensitivity^32^.

### Deformation behaviour as a function of *d*/*s*

**Figure 3:**
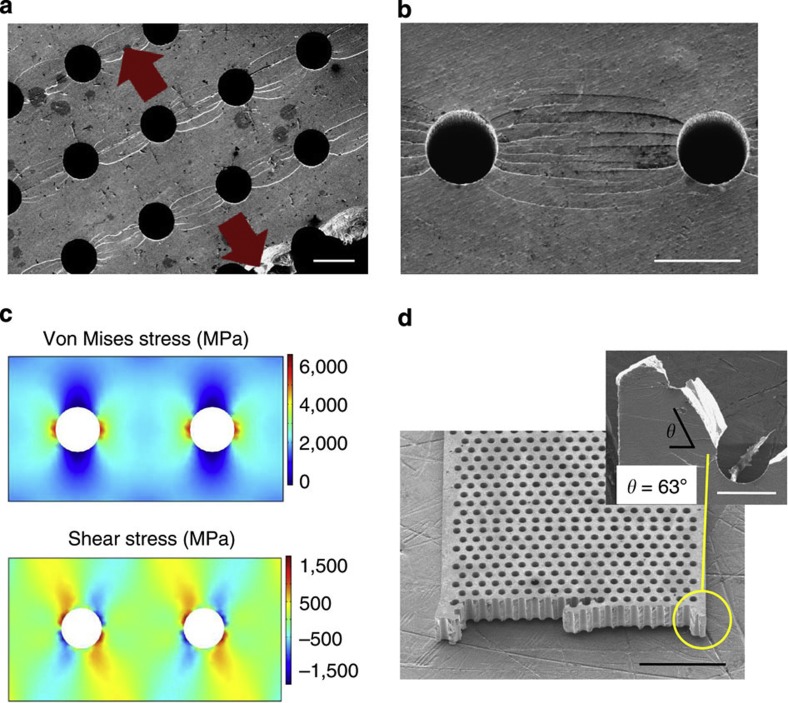
Microscopic analysis of the deformation mechanism in MG heterostructures. (**a**) Global tensile ductility originates from the formation of multiple shear bands throughout the sample (scale bar, 50 μm). (**b**,**c**) Shear concentrations at the pores initiate the formation of shear bands, and redirect their propagation towards the nearest neighbour pore (scale bar, 100 μm). (**d**) Fracture of the MG heterostructure occurs perpendicular to the loading direction (scale bar, 1 mm). Fracture is redirected at the sample’s edges, following the shortest distance between the pores (inset—scale bar, 100 μm).

In order to understand the microscopic origin of the MG heterostructures’ tensile ductility, we carried out scanning electron microscopy imaging (Fig. 3). For the *d*=*s*=50 μm heterostructure, which was deformed until failure (*ε*_f_≈7.5%), a large number of shear bands form between the pores throughout the entire gauge section (Fig. 3a). These shear bands carry the plastic deformation, and their large number results in observed global tensile ductility. Shear bands initiate at pores, where they follow the direction towards the neighbouring pore, in a similar pattern as observed in double notched MG samples^33^ (Fig. 3b). Finite element modelling (FEM) simulations show that, as deformation proceeds, the complex stress field (Fig. 3c) redirects shear bands towards neighbouring pores. Fracture occurs typically along the shortest distance between pores, which is for the considered sample dimensions, perpendicular to the uniaxial loading direction (Fig. 3d). At the edges of the sample, fracture chooses a different path by following the shortest distance in the diagonal pore direction (inset in Fig. 3d).

It should be mentioned that the magnitude of the stress concentrations depends also on the shear modulus of the second phase, where a second phase with significantly different shear modulus than the matrix causes high stress concentrations. From the FEM results, we conclude that the lowest stress concentrations are present for a second phase with a modulus similar to the matrix. However, in this situation, elastic strain values as shown in Fig. 2b cannot be achieved.

**Figure 4:**
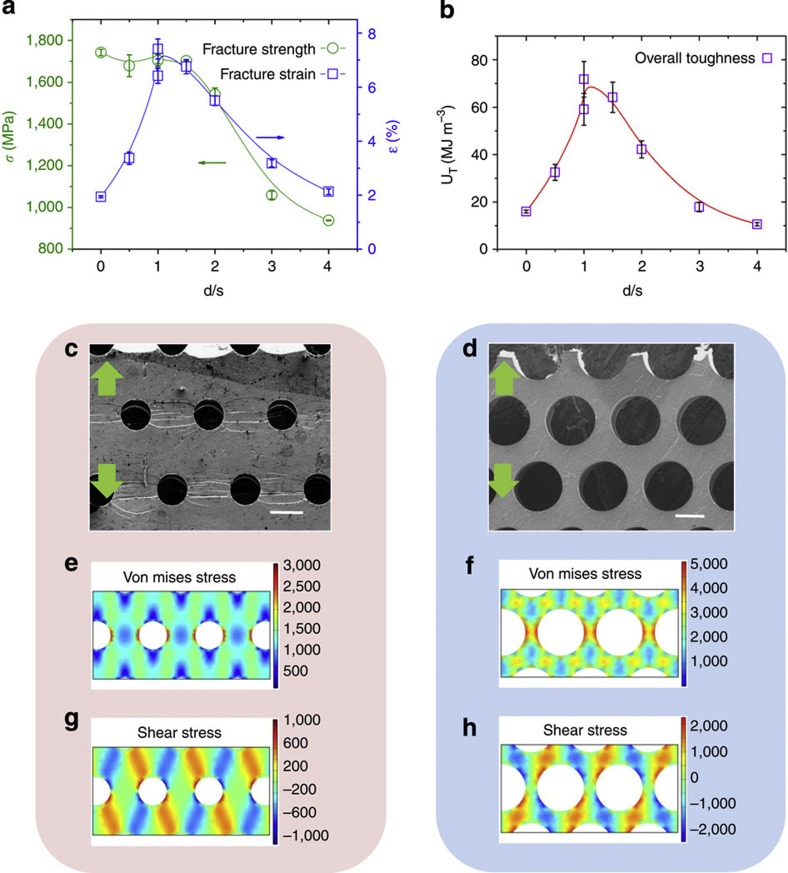
Effect and origin of *d/s* on mechanical properties in MG heterostructures. (**a**) Fracture stress and fracture strain as a function of the ratio of diameter to spacing of the pores (*d/s*). (**b**) Toughness (*U*_T_) versus *d/s* exhibits a pronounced maximum at *d/s*≈1. Error bars represent the s.d. of the mean values from at least three test samples. (**c**) Corresponding scanning electron microscopy image of MG heterostructures with *d/s*=1 showing multiple shear bands (scale bar, 50 μm). (**d**) For an applied average stress of 1,000 MPa, the spatially confined stress concentrations between the pores perpendicular to the loading direction are lower compared with heterostructures with *d/s*=4, hence multiple shear bands form without crack formation. (**e**–**g**) Shear bands initiate at the shear stress concentration regions adjacent to the pores. (**f**–**h**) For *d/s*=4, stress concentration pathways between the pores already exceed *σ*_y_ for the same average applied stress, resulting in early fracture from only a few shear bands (scale bar, 50 μm).

The experimental results (Fig. 2a) reveal the pores’ diameter to spacing ratio (*d/s*) as the critical factor that controls the mechanical behaviour of MG heterostructures (Fig. 4a). Fracture strength decreases with increasing *d/s*. Fracture strain, on the other hand, does not increase monotonously, but instead, exhibits a maximum at *d/s*≈1. The maximum in *ε*_f_ originates from an increase in the elasticity and plasticity of the AB-stacking heterostructure with increasing *d/s*, which is eventually overcompensated by rapidly increasing stress concentration between pores. As a consequence, the toughness of the MG heterostructure 
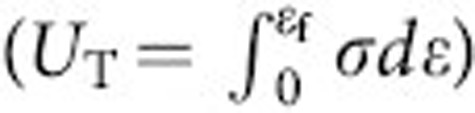
 exhibits a maximum at *d/s*≈1 of *U*_T_≈80 MJ m^−3^, which is ~5 times higher than for the monolithic Zr_35_Ti_30_Cu_7.5_Be_27.5_ MG of *U*_T_=16 MJ m^−3^ (Fig. 4b). It is noteworthy to state that the change in *d* and *s* have negligible effect on fracture stress and strain for the same *d/s*=1 sample. Therefore, we concluded that *d/s* is the prominent factor in controlling the stress distribution between the pores, and thereby, the fracture stress and strain.

To reveal the microscopic origin of the dependence of *U*_T_, *ε*_f_ and *σ*_f_ on *d/s*, we investigated the shear band propagation and distribution across the pores. Specifically, we characterized shear band patterns for different *d/s* ratio, and compared those with stress fields, which we determined through FEM. For *d/s*=1, a large number of shear bands form throughout the sample (Fig. 4c). FEM analysis indicates that resultant von Mises stress in the middle region between the pores perpendicular to the loading direction of ~800 MPa is lower than the applied stress of 1,000 MPa. Stress concentrations are confined to small, non-percolating regions adjacent to the pores (Fig. 4d). For *d/s*=4, only a very small number of shear bands form before fracture (Fig. 4f). Thus, the resulting toughness of the heterostructure is low (Fig. 4b). FEM results reveal that resultant von Mises stresses for *d/s*=4 are significantly higher (~3,200 MPa) for an applied stress of 1,000 MPa, and stress concentrations overlap between pores (Fig. 4g). As a consequence, MG heterostructures with *d/s*=4 yield at a lower applied load level, and hence, the total amount of plastic deformation until fracture is much smaller (Fig. 2d). As a result, only a small number of shear bands form before they develop into cracks.

### Effect of embrittlement and crystallization

**Figure 5:**
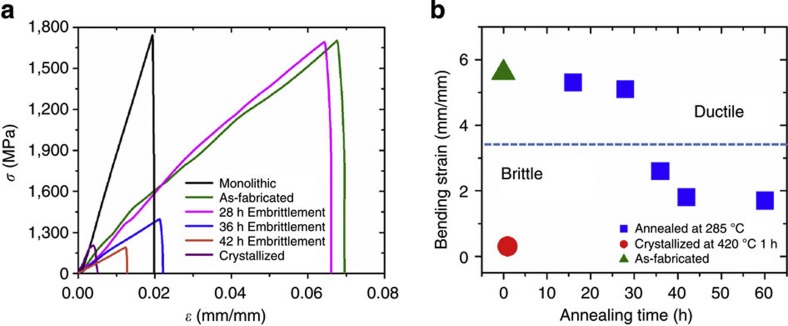
Influence of sub-*T*_g_ annealing and crystallization on the ductility of heterostructures. (**a**) For samples that are completely structurally relaxed at 558 K, as well as for crystallized samples, ductility decreased dramatically. (**b**) Bending test reveals a significant decrease in plasticity for annealing times exceeding 28 h at 558 K.

In addition studying the effect of pore spacing on the MG-heterostructures’ performance, we also varied the properties of the matrix material, and thereby, its critical crack length. An effective strategy to vary the critical crack length is to structurally relax (embrittle) the MG through thermal annealing^11^,^13^,^34^,^35^,^36^,^37^,^38^,^39^. We fabricated tensile samples out of Zr_35_Ti_30_Cu_7.5_Be_27.5_ MG former with *d/s*=1.5, which were annealed at 558 K, ~25 K below its calorimetric glass transition temperature. The degree of structural relaxation is determined through enthalpy recovery^40^ (not shown here), and its effect on mechanical behaviour through bending tests (Fig. 5b). Tensile characterization shows that strain to failure and *U*_T_ are dramatically affected by annealing (decrease in *a*_c_) and crystallization (Fig. 5a). The completely structurally relaxed MGs for 36 h and longer, as well as the crystallized MG former approximate ideal brittle behaviour with *K*_IC_<5 MPa m^1/2^ (refs 41, 42), and exhibit a critical crack length below 1 μm. In such MG heterostructures, *a*_c_<*s*, and as a consequence, the overall strain decreases to the elastic limit of ~2%. Even though *K*_IC_ is not directly quantified in this work, it can be concluded from Fig. 5b that for the as-cast and 28 h annealed heterostructures *a*_c_>*s*, hence shows large tensile ductility.

## Discussion

The aim of our approach is to identify and understand the mechanisms that result in tensile ductility in MG heterostructures such as foams, cellular heterostructures and, to a limited extent, composites. The strength and purpose of our method is the ability to precisely vary individual microstructural features completely independent, and determine the effect of such isolated variations on the mechanical response. As such, our method is not intended as a scalable method to fabricate MG heterostructures for applications, but as a method to identify and understand the effectiveness of some MG heterostructures to guide design and synthesis of MG foams, cellular structures and composites.

Our results revealed three critical aspects of a MG heterostructure, which control mechanical behaviour. One is the difference between the second phase spacing and the critical crack length of the MG, *s*−*a*_c_. For *s*−*a*_c_<0, shear bands that form between pores do not grow to a length where they transform into a crack. As a consequence, global tensile ductility can be achieved through the formation of multiple shear bands. For *s*−*a*_c_>0, a shear band can develop into a crack before strain energy releases into the pore and, consequently, only one or a few shear bands form before crack formation, which impairs tensile ductility. Our finding has previously been broadly confirmed in MG heterostructures with a soft second phase, that is, G_2nd_<G_matrix_, such as foams, cellular structures and composites^2^,^9^,^20^,^21^,^23^,^24^,^25^; nevertheless, up to date, it has not been proven owing to experimental limitations.

However, the *s*−*a*_c_ criterion alone is insufficient in predicting the performance of MG heterostructures with a soft second phase owing to the effect of stress concentrations on the deformation mechanism. In addition to *s*−*a*_c_, we identified the ratio of second phase size to spacing as a critical feature for toughness and tensile ductility optimization. Maximum toughness is achieved for *d/s*≈1, which corresponds to a density of ~50%. This value for *d/s* exhibits the best compromise between enhanced stress concentrations, which limits plastic deformation, and thin interporous regions, which enhance plasticity in cellular structures.

Our design criteria is suggested to be readily applicable to cellular MGs, whereas the applicability for the effective design of MG composites is limited, which is owing to the complex nature of MG composites, and the mechanisms that toughen them. Experimental findings suggest influences on the toughening mechanism of shape and volume fraction of the second phase, interfacial strength and toughness, softness of the second phase reflected in shear modulus, and even dislocation and twinning in the second phase. Our finding of the drastically different mechanical response between MG heterostructures with AB and AA stacking exemplifies the importance of the second phase morphology and the overall complex nature of the toughening mechanism in MG composites.

Furthermore, the softness of the second phase has been broadly suggested to have a crucial role in the toughening mechanism^43^. Even though the pores in the MG heterostructure have a smaller shear modulus than the matrix, which has been recognized to be a common feature of effective toughening mechanisms, it is not clear if pores can be considered as a soft second phase, where their shear modulus is significantly lower than *G*_matrix_, and they lack an interface.

We argue that the effectiveness of MG heterostructures also depends on the size (cross-section width perpendicular to the loading direction) of the sample, *w*, in comparison with *s*. Such a size effect originates from the fact that shear bands do not support tensile forces during the actual shearing event. As the cross-sectional area supporting a tensile load in samples, where *w* is not much larger than *s,* is significantly reduced when one shear band forms, the applied load results in exceeding *σ*_y_ throughout the sample. Therefore, small samples with *w* comparable to *s* fail catastrophically along one shear band. In large samples, *w*>>*s* (assuming *s*−*a*_c_<0), upon formation of a shear band between two neighbouring pores, stresses between most of the other pores typically do not exceed *σ*_y_ as the stress increases with the area reduction, *w*–*s*, owing to the initial shearing event being small. Our finding also explains why all reported MG composites, which are exhibiting tensile ductility, obey *w*>>*s*.

Our results also reveal the possibility to design elasticity through the elasticity and the modulus of the second phase in MG heterostructures. As shown in Fig. 2b, we have demonstrated up to 3.9% of elastic strain for the heterostructure with *d*=100 μm and *d/s*=2, and an AB-stacking order. Morphology in MG foams and composites is AB-like; however, in MG foams and composites, an increase in elasticity compared with the monolithic MG has not been reported yet. For MG composites, this is owing to the properties of the second phase, which exhibits low elasticity (typically below 0.5% and comparable modulus to the MG matrix), thereby preventing increased heterostructure elasticity. MG foams with controlled features have not been tested in tension yet.

In summary, we identify the most effective heterostructure to result in tensile ductility and toughness in MGs. Such structure features a spacing of the second phase, which coincides with the critical crack length of the MG and with the second phase size. In addition, we find that heterostructure morphology is crucial and the size of the sample should be large compared with the second phases’ spacing.

## Methods

### MG alloy production

Zr_35_Ti_30_Cu_7.5_Be_27.5_ alloy was prepared by melting the elements with purity better than 99.99% in an arc melter. To prepare an amorphous rod, the ingot was sealed in a quartz tube under vacuum, which was then heated above its liquidus temperature for 5 min, and subsequently quenched in water. X-ray diffractometer (XRD-6000 Shimadzu) and differential scanning calorimeter (Perkin Elmer Diamond DSC) confirmed the amorphous nature of the Zr–MG rods.

### Si mould fabrication

We design MG heterostructure patterns using AutoCAD 2011 and Layout Editor. The drawing layout was transferred to a photomask maker (Heidelberg DWL-66 Laser Mask Writer), and the photomask is generated using direct laser beam writing. A thin layer of photoresist (8-μm AZ 5214 coating) on 650-μm-thick Si wafer was patterned by conventional photolithography using EVG 620 Contact/Proximity Mask Aligner. The etch cycle for DRIE processing (Bosch etch) was carried out under 12 s, 130-s.c.c.m. SF_6_, 13-s.c.c.m. O_2_, 600-W coil power, 12-W platen power and 26 mtorr pressure, and the passivation cycle was performed under 8 s, 85-s.c.c.m. C_4_F_8_, 600-W coil power, 0-W platen power and 15 mtorr pressure. The overall etch rate was 4 μm min^−1^, where the final depth of the Si moulds was measured to be around 400±20 μm. Residual resist was subsequently stripped from the surface using an etchant (PRS 3000), and cleaned by soaking first in acetone, then in isopropanol and finally in deionized water. The Si wafer was finally dried with N_2_ under a pressure of 2 × 10^5^ Pa.

### MG heterostructure fabrication

MG discs cut from the amorphous cast rods were pre-pressed to a thickness of around 800 μm using custom-built heating system in conjunction with Instron 5569 tensile testing machine (50 kN maximum load capacity). Using thermoplastic-based compression moulding, MG heterostructures were fabricated from Zr_35_Ti_30_Cu_7.5_Be_27.5_ MG former at 425 ˚C for 60 s under a pressure of 50 MPa using the above described setup. The extra MG layer remaining on the top surface of the silicon was later removed by grinding and polishing using Buehler Metaserv 250 Grinder-Polisher. The MG tensile heterostructures are subsequently taken out of the mould by Si mould etching in KOH for 60 min at 100 °C. The amorphous heterostructure of the MG honeycomb was later confirmed by thermal and structural analysis using DSC and XRD, respectively. Mechanical characterization of the MG heterostructures was conducted using Instron 5543 Tensile Tester (1 kN maximum load capacity) under quasi-static conditions (strain rate of 0.001 s^−1^). Simultaneous image recording is conducted by Keyence Digital Microscope VHX-500F. Scanning electron microscopy imaging (Model: Hitachi SU-70) was subsequently utilized to determine the fracture pattern and the distribution of shear bands between pores.

### Finite element simulation

COMSOL Multiphysics Engineering Simulation Software was utilized to conduct structural element analysis for AB-stacking MG heterostructures. Linear elastic material model was used to approximate the mechanical behaviour of the Zr–MGs. The thickness of the specimen was set to be 300 μm. The surface was divided into refined triangular meshes, and an applied stress of 1,000 MPa is evenly distributed among the pores. Von Mises stress, shear stress and elastic strain around the pores were measured and compared with AA-stacking MG heterostructure, and other AB-stacking MG heterostructures with a second phase such as polymer, soft metal and graphite.

### Bending test

Rectangular beams of 0.65±0.05 mm thick were prepared from the standard samples. All sides of the beams were mirror polished, and the corners were rounded to avoid stress concentration during bending tests. Beams were bent over mandrels with decreasing radius from 30–5.5 mm with corresponding strains of 1.2–6% under constant compressive strain rate of 0.005 s^−1^ using Instron Testing Machine. This range spans the elastic to the plastic strain region, which enables to observe changes in the bending ductility with high accuracy. XRD spectrum and diffraction scanning calorimetry thermogram confirms structural differences between as-fabricated and sub- and above-T_g_ annealed MG heterostructures (not shown here).

### Heat treatment of MG heterostructures

Zr_35_Ti_30_Cu_7.5_Be_27.5_ MG heterostructures were annealed for different time intervals at 285 °C for sub-Tg embrittlement, whereas the crystallization was achieved by heating the MG heterostructure to 420 °C (above-Tg) for 1 h using Vulcan Box Annealing Furnace (Model No: 3–550). Embrittlement can be studied in a wide range of MG alloys. DSC and XRD measurements were conducted to determine the effect of different annealing processes on thermal and structural properties.

## Author contributions

J.S. and B.S. designed the research. B.S. fabricated the structures, conducted the experiments and data analysis, and composed the manuscript. J.S. supervised the research and preparation of the manuscript.

## Additional information

**How to cite this article:** Sarac B. and Schroers J. Designing tensile ductility in metallic glasses. *Nat. Commun.* 4:2158 doi: 10.1038/ncomms3158 (2013).
